# Functional Movement Disorder to Huntington’s Chorea: Unmasking the Underlying Condition in a Patient Initially Diagnosed with Anxiety Disorder

**DOI:** 10.1192/j.eurpsy.2025.1245

**Published:** 2025-08-26

**Authors:** B. Marsap, M. H. Aksu

**Affiliations:** 1 Psychiatry, Gazi University Hospital, Ankara, Türkiye

## Abstract

**Introduction:**

Huntington’s disease (HD) is a hereditary neurodegenerative disorder characterized by motor, cognitive, and psychiatric symptoms. Initially, the patient’s involuntary movements were attributed to anxiety-related restlessness and psychomotor agitation, leading to a diagnosis of anxiety disorder. During hospitalization, the patient was referred to neurology for his movements, where functional movement disorder was considered. However, due to worsening cognitive decline, apathy, and the psychiatrists’ suspicion that the movements were not functional, further investigations were conducted. Cranial MRI, followed by a dementia protocol MRI, revealed caudate nucleus atrophy, leading to the diagnosis of HD.

**Objectives:**

To illustrate the diagnostic challenges in identifying Huntington’s disease in a patient initially misdiagnosed with anxiety disorder and functional movement disorder.

**Methods:**

A 58-year-old male presented with anxiety and involuntary movements, leading to initial diagnoses of anxiety disorder and functional movement disorder. As symptoms progressed—including worsening involuntary movements and cognitive decline—a comprehensive reassessment was conducted. This included neurological examination, neuroimaging, neuropsychological testing, and genetic testing via blood test for CAG repeat analysis.

**Results:**

Cranial MRI revealed bilateral caudate atrophy and dilated lateral ventricles, consistent with Huntington’s disease. Neuropsychological assessment showed significant impairments in verbal learning, memory, and executive function. The patient was treated with venlafaxine 150 mg/day, mirtazapine 30 mg/day, and haloperidol 6 drops/day, leading to significant improvement in anxiety symptoms and a reduction in chorea.

**Conclusions:**

This case underscores the importance of considering neurodegenerative disorders such as Huntington’s disease in the differential diagnosis of patients presenting with psychiatric symptoms and involuntary movements. Early identification, comprehensive assessment, and a thorough understanding of HD’s clinical presentation are crucial for appropriate management.

**Disclosure of Interest:**

None Declared

